# Fathers’ eye colour sways daughters’ choice of both long- and short-term partners

**DOI:** 10.1038/s41598-018-23784-7

**Published:** 2018-04-03

**Authors:** Paola Bressan, Valeria Damian

**Affiliations:** Dipartimento di Psicologia Generale, Università di Padova, 35131 Padova, Italy

## Abstract

In several species, mate choice is influenced by parental features through sexual imprinting, but in humans evidence is scarce and open to alternative explanations. We examined whether daughters’ preference for mates with light vs dark eyes is affected by the eye colour of parents. In an online study, over one thousand women rated the attractiveness of men as potential partners for either a long- or a short-term relationship. Each male face was shown twice, with light (blue or green) and with dark (brown or dark brown) eyes. Having a light-eyed father increased the preference for light-eyed men in both relationship contexts. Having light eyes increased this preference too, but only when men were regarded as potential long-term companions. Asymmetrically, in real life, father’s eye colour was the only predictor of partner’s eye colour; own colour was irrelevant. Mother’s eye colour never mattered, affecting neither preferences nor real-life choices. The effect of paternal eye colour was modulated by the quality of the relationship between father and daughter, suggesting (flexible) sexual imprinting rather than a simple inheritance of maternal preferences. Our data provide evidence that in humans, as in birds and sheep, visual experience of parental features shapes later sexual preferences.

## Introduction

Across a wide variety of species—from fish to birds and mammals—mate choice is influenced by the early experience of parental features, a mechanism known as sexual imprinting^1,2^. Cues are often visual. For example, when nanny goats are used as foster parents for male lambs, the lambs grow into adults that prefer to socialise and mate with goats, rather than with sheep. What they are imprinting on is facial features, as shown by the fact that, given the option to spend their time close to a picture of the face of either a female sheep or a female goat, they clearly prefer the goat’s^3^. Here the male lambs “imprint” upon the maternal figure, and in some (though not all) species this turns out to be a defining feature of sexual imprinting: sons tend to imprint positively on mothers^4^ and daughters on fathers^5^.

In humans, considering the dubious propriety of carrying out similar interventions, evidence on whether parental characteristics shape mate preference is much less direct. Echoing the way mother goats affect male lambs’ future choices, children of ethnically different parents are more likely to marry someone of the same ethnicity as their opposite-sex, rather than same-sex, parent^6^. Women born to older fathers tend to marry older men^7^ and women with older parents are more attracted to older faces than are women with younger parents—whether the facial signs of age are natural^8^ or the result of digital manipulation^9^.

Several studies have tested whether people’s parents and (actual or preferred) partners tend to be more similar in facial traits than randomly selected individuals, but results are mixed (as reviewed in^10–12^). Of course, facial appearances are affected by sex, age, and physical condition. They result from the interaction of a large number of traits and do not lend themselves readily to measurement. A viable alternative is to measure resemblance in one simple, conspicuous facial feature. Eye colour stands as an ideal candidate. It is lodged in the most interesting part of the face, exceptionally salient in close interactions, unaffected by sex, age and physical condition, and highly (98%^13^) heritable. From a genetic^14^ (and arguably, also perceptual^15^) viewpoint, the most meaningful distinction between eye colours may be dark (brown) vs light (nonbrown: blue, grey, or green). In fact, although eye colour is polygenic, a single variation in the HERC2 gene determines whether the human ancestral brown eye colour is on or off^14^. If one of the two variants is inherited, eyes will be brown; if the other is, eyes will be blue or green or variations thereof (depending on other genes and modifiers).

In any parental-face mould that the young build for future reference, eyes must feature prominently^16^ and their colour is likely to be attached to them. There is indeed some indication that the iris colours of people’s partners and opposite-sex parents tend to be more similar than expected by chance. A weak association between father’s and boyfriend’s eye colours (blue vs brown) has been reported in a sample of 217 young women^17^. In an internet survey (394 men, 303 women), small but significant correlations emerged between the eye colours of men’s partners and mothers, women’s partners and fathers^18^.

Imprinting on her father may allow a girl to form a template of what her future mate should look like. If so, two possibilities present themselves. Because he is a man, the father could be a model for a sexual partner in general, either short- or long-term. Or, because he is a husband and father, he could be a model for a husband and father, that is, for a long-term companion. The probability that he is taken as a model may in fact depend on his observed competence as a husband and father—especially as a father, a competence that the daughter is in an excellent position to judge.

In the study presented here we sought to determine, in a large single-ethnicity sample, whether eye colour preferences in women are biased by the eye colour of parents. In an online study, 1233 Italian women were shown different pairs of identical male faces with light vs dark eyes and rated them for attractiveness in two potential relationship contexts: a sexual encounter and a long-term commitment. Together with other pieces of demographic information, women reported their own, their parents’, and their partner’s eye colour, and evaluated the quality of their relationship with each parent.

## Methods

### Participants

Participants were 1233 women (mean and median age = 23 years, range = 18–56 years). Of these, 687 reported having a partner. The study was conducted online and participants were recruited mostly via links posted on Italian universities’ online social networks. The experimental protocol was approved by the Psychological Research Ethics Committee of the University of Padua and was in accordance with the relevant guidelines and regulations. Informed consent was obtained from all participants.

### Materials and procedure

**Figure 1:**
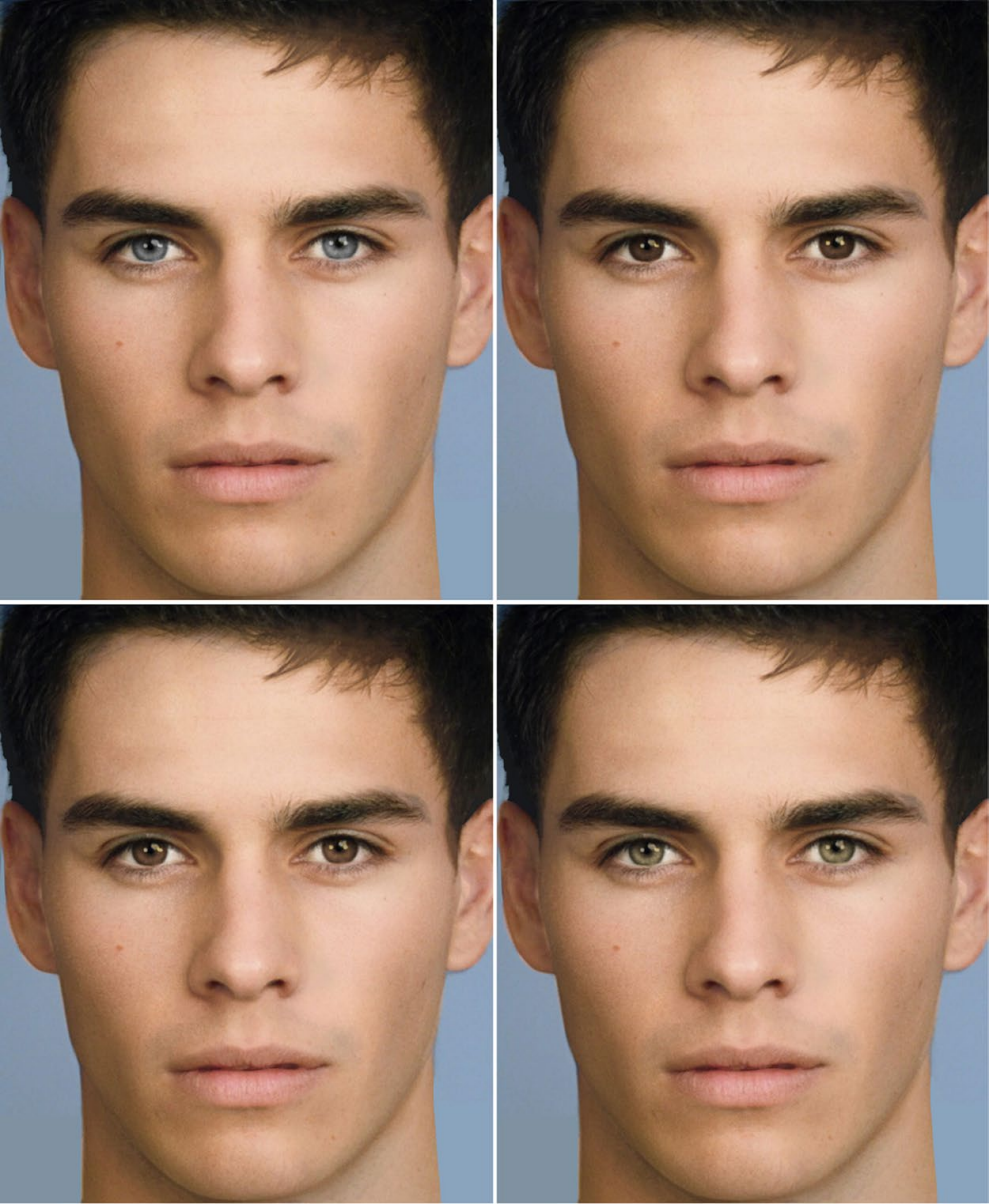
Example stimuli. Stimuli were pairs of identical faces with light vs dark eyes. Top: blue-dark brown pair. Bottom: brown-green pair. The face depicted here has been created digitally and is used for illustration purposes only; the study presented photographs of real people. Image copyright by Paola Bressan.

We selected and digitally modified 10 colour photographs of attractive men’s faces—Caucasian models or actors little known in Italy. The photos were imported into PortraitPro, a professional software for portrait retouching. We created four versions of each face by changing the eye colour into natural shades of blue, green, mid brown, and dark brown—while retaining the textural properties of the iris and the original highlights and shadows. Each set of four images was combined into two pairs (Fig. 1): blue-dark brown (side by side, with blue on the left) and brown-green (side by side, with green on the right). Thus, for each face we had one light-dark combination (blue-dark brown) of higher contrast and one (brown-green) of lower contrast.

The first pair was assigned to one virtual album and the second to another, and this procedure was repeated for all stimuli in alternation, so that each album contained the same 10 men, but the 5 men presented as “blue-dark brown” in one album were featured as “brown-green” in the other. Thus, participants saw 10 different pairs of identical faces with light vs dark eyes, with the left-right position of light eyes counterbalanced across stimuli. Each set of 10 stimuli was presented twice, once in a short-term relationship context and once in a long-term one. The order of contexts (short-term first, long-term first) was counterbalanced across participants by creating two separate copies of each album, for a total of four albums. The distribution of participants into albums was obtained by dividing the alphabet into four parts, corresponding to four separate links, and having each woman click on the letter group her surname belonged to.

Each face pair was accompanied by three questions. The first was, “If you were looking for a long- (short-) term relationship, which of these two people would you prefer?”. Possible responses, presented side by side with radio buttons that could be clicked on, were “the one on the left” and “the one on the right”. The second and third questions were, “For a long- (short-) term relationship, how attractive do you find the person on the left?” and “For a long- (short-) term relationship, how attractive do you find the person on the right?”, each followed by a 0–10 scale going from “not at all attractive” to “very attractive”.

The definitions of long-term and short-term relationships (similar to Perrett *et al*.’s^9^,) were given at the beginning of the corresponding block and were formulated as follows: “By a long-term relationship, we mean someone you may consider leaving your current partner to be with, or with whom you may like to create a stable relationship, leading to cohabitation or marriage, in case of breakup with a current partner”; “By a short-term relationship, we mean a single date accepted on the spur of the moment, an affair within a long-term relationship, or a one-night stand”.

After completion of this part of the study, participants answered a few questions about themselves, their mother, their father, and their current partner if they had one. The eye colour that best described the person being judged was selected by a list; choices were “dark brown”, “brown”, “hazelnut (very light brown)”, “green”, “grey”, “blue”, and “other” (with the option to specify). (“Hazelnut” is the translation of the Italian “nocciola”, which when used as an eye-colour label indicates a very light brown.)

We collected two separate measures of participants’ relationship quality with each parent. The first was a direct assessment (“How would you evaluate your affective relationship with her [him]?”) on a 0–10 scale from “very bad” to “very good”. The second was a recollection of rejection behaviours displayed by the parent during the participant’s childhood. This was measured via three representative items of the Rejection Scale of the s-EMBU, a measure of adults’ perception of their mother’s and father’s rearing behaviour^19^. These were: “I was treated as the ‘black sheep’ or ‘scapegoat’ of the family”, “[My parent] would punish me hard even for small things”, and “It happened that [my parent] was cold or angry with me without letting me know the reason”. The frequency of each behaviour was estimated on a 4-point scale going from “no, never” (coded as 0) to “yes, very often” (coded as 4). Hence, maternal and paternal rejection scores could range from 0 to 12.

### Data analysis

For correlational analyses, eye colour was coded on a 5-point scale from light to dark (1 = blue, 2 = green, 3 = very light brown, 4 = brown, 5 = dark brown). This corresponds to a natural continuum in the amount of melanin found in the iris (e.g.^20^); similar ordinal classifications, with variable numbers of points, have been used before^18,21^. All eye colours that did not unambiguously belong to one category, such as “amber” or “greenish brown”, or that were exceedingly rare, such as “grey” (1%), were left out.

For analyses on preferences for light-eyed (blue or green) vs dark-eyed (brown or dark brown) men, eye colour was instead coded as “light” (categories “blue” and “green”) and “dark” (categories “brown” and “dark brown”). Participants with very-light-brown eyes were therefore excluded from this set of analyses.

For all analyses involving parents and daughters, we considered only women who reported having cohabited with the relevant parent for longer than 1 year. The rationale was that, if the parent died or left when the daughter was 1 year old or younger, there would have been little or no opportunity for the daughter to imprint on the parent. This led to exclusion of 2 participants for analyses involving mothers and 18 participants for analyses involving fathers. (The pattern of results was the same with and without these exclusions.)

Of the 1233 women that completed the study, 7 (0.6%) reported being homosexual and 9 (0.7%) failed to disclose their sexual orientation. Because analyses with and without these individuals gave virtually identical results, here we conservatively present the results for all participants.

On virtual-partner data, we used point-biserial correlations to test the relationship between own, maternal, paternal eye colour (light vs dark) and preference for light-eyed virtual partners. Where appropriate, correlations were also performed with the effects of own eye colour partialled out. Significant relationships were further examined via ANOVAs conducted both across the short-term and long-term relationship contexts (repeated-measures) and separately for each of them (univariate).

On real-partner data, we used bivariate correlations to test the relationship between own, maternal, paternal, and partner’s eye colours as coded on the 5-point scale from light to dark; because of the ordinal nature of this scale, we chose nonparametric Spearman’s rho (*r*_S_). Where appropriate, correlations were also performed with the effects of own eye colour partialled out. Additionally, we carried out chi-square tests on the frequencies of father-partner eye-colour pairs to determine whether some combinations were observed more (or less) often than would be expected by chance.

All quoted probabilities are two-tailed and rounded to one significant digit^22^ (i.e., the first non-zero digit after the decimal point) except where this would create ambiguity about statistical significance (i.e., when *p* is rounded, up or down, to 0.05), in which case the second significant digit is indicated too.

### Data availability

The data that support the findings of this study are publicly available at figshare.com/s/a4a774a524fa16e58797.

## Results

### Virtual-partner choice

For each participant and context (short-term, long-term), we computed the preference for light-eyed men as the number of times the light-eyed face was preferred to the dark-eyed face relative to the total number of preferences. This proportion can vary from 0 (light-eyed face is never chosen) to 1 (light-eyed face is always chosen).

In both relationship contexts, women’s preference for light-eyed men was predicted by having a light-eyed father (long-term: *r* = 0.13, *p* < 0.0001, *N* = 1038; short-term: *r* = 0.12, *p* = 0.0002, *N* = 1037), but not by having a light-eyed mother (long-term: *r* = 0.01, *p* = 0.7, *N* = 1032; short-term: *r* = 0.01, *p* = 0.8, *N* = 1033). Significant results remained significant, and nonsignificant results remained nonsignificant, if own eye lightness was controlled for (via partial correlations).

On preferences for light-eyed men we ran a repeated-measures ANOVA, with a within-subject factor of relationship context (long-term, short-term) and between-subject factors of own and paternal eye colour (light, dark). Paternal eye colour was significant as a main effect, *F*(1, 869) = 10.9, *p* = 0.001; crucially, it did not interact with context, *F* < 1. Own eye colour, instead, did, *F*(1, 869) = 7.5, *p* = 0.006. As shown by two additional ANOVAs run separately for the two contexts, being light-eyed increased the preference for light-eyed men as long-term partners, *F*(1, 870) = 9.9, *p* = 0.002, but not as short-term ones, *F* < 1. On the other hand, having a light-eyed father increased the preference for light-eyed men as *both* long-term partners, *F*(1, 870) = 6.1, *p* = 0.01, and short-term ones, *F*(1, 869) = 10.1, *p* = 0.002.

The influence of father’s light eyes on the preference for light-eyed men as long-term partners was modulated by how much daughters had felt rejected by their father during childhood. If participants are subdivided into tertiles according to their paternal rejection score (0–3; 4–5; 6–12), the correlation between paternal light eyes and preference for light eyes varies accordingly. Lowest tertile: *r* = 0.18, *p* = 0.0007, *N* = 342; middle tertile: *r* = 0.16, *p* = 0.002, *N* = 410; highest tertile: *r* = 0.02, *p* = 0.8, *N* = 286. The lowest and highest tertile correlation coefficients are significantly different, *Z* = 2.1, *p* = 0.04, two-tailed. An effect in the same direction emerged for short-term preferences (lowest tertile: *r* = 0.20, *p* = 0.0003; highest tertile: *r* = 0.04, *p* = 0.5; *Z* = 2.0, *p* = 0.049, two-tailed). Subdivision into quartiles rather than tertiles, or into tertiles of paternal relationship quality instead of paternal rejection, gave similar results.

**Figure 2:**
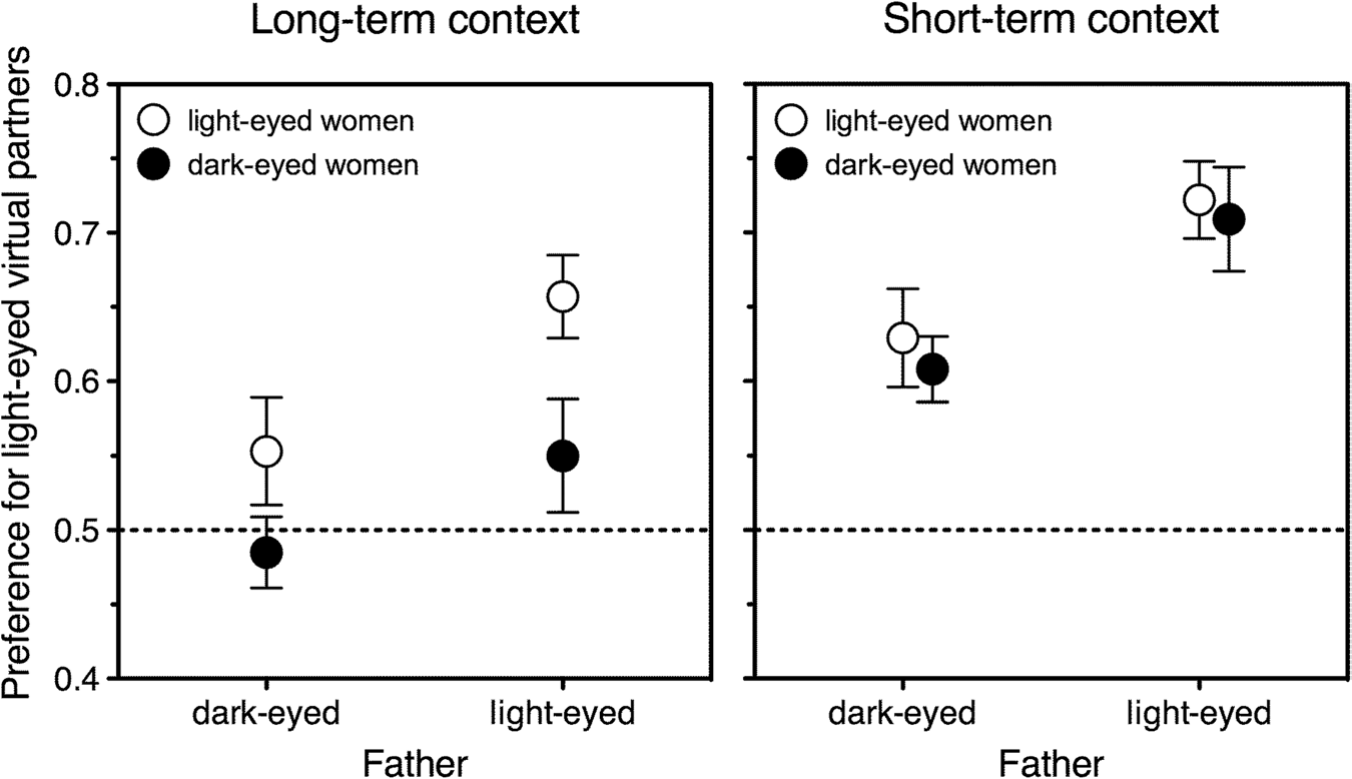
Mean preference for light-eyed men as potential long-term (left panel) and short-term (right panel) partners, as expressed by women in the lowest tertile of paternal rejection. Choices are plotted as a function of own and father’s eye lightness. Values higher than 0.5 (chance level) represent a preference for light-eyed over dark-eyed men. Error bars indicate one standard error of the mean. Left panel: The effect of father’s eye lightness (compare symbols on the left with symbols on the right) is significant; the effect of own eye lightness (compare open symbols with filled symbols) is also significant. Right panel: Only the effect of father’s eye lightness is significant.

To explore the effect of paternal rejection we reran the repeated-measures ANOVA on the women in the lowest and highest tertile of paternal rejection only, so as to include an additional between-subjects factor of paternal rejection (low, high). Own eye lightness did not interact with paternal rejection, *F* < 1, but father’s eye lightness did, *F*(1, 525) = 5.2, *p* = 0.02. As revealed by separate ANOVAs, this interaction was due to the fact that the light eyes of fathers by whom daughters had most felt rejected did not affect their choices, *F* < 1, whereas the light eyes of fathers by whom daughters had least felt rejected did, *F*(1, 295) = 19.3, *p* < 0.0001. Figure 2 depicts the preferences of the latter group of women.

Inspection of Fig. 2 also suggests that, even in the scenario where paternal eye lightness matters most, women’s preferences tend to be asymmetrical—i.e., women like light eyes better than dark eyes. Being irrelevant to the topic of sexual imprinting, this result will be explored in depth at some future time and is not discussed further in this article.

### Real-partner choice

Considering mothers, fathers, daughters and partners, we had 705 people with blue, 897 with green, 416 with very-light-brown, 1388 with brown, and 742 with dark-brown eyes, for a total of 4148 individuals.

Own eye colour, as coded on the 5-point scale from blue to dark brown, correlated positively with both maternal (*r*_S_ = 0.36, *p* < 0.0001, *N* = 1106) and paternal (*r*_S_ = 0.41, *p* < 0.0001, *N* = 1088) eye colours. This was to be expected, given that eye colour is an inherited trait. We found no trace of positive assortative mating: no significant correlation between maternal and paternal eye colours (*r*_S_ = −0.06, *p* = 0.055, *N* = 1115) and no significant correlation between own and partner’s eye colours (*r*_S_ = 0.005, *p* = 0.9, *N* = 615).

Mirroring virtual-partner preferences, in real life there was no significant correlation between mother’s and partner’s eye colours, *r*_S_ = −0.04, *p* = 0.3, *N* = 626. Mirroring preferences, again, father’s and partner’s eye colours correlated positively and significantly, *r*_S_ = 0.14, *p* = 0.0005, *N* = 599 (controlling for own eye colour: *r*_S_ = 0.15, *p* = 0.0002, *df* = 589). Results were the same if eye colour was coded as light vs dark, like in virtual-partner choices, rather than on the 5-point scale.

**Figure 3:**
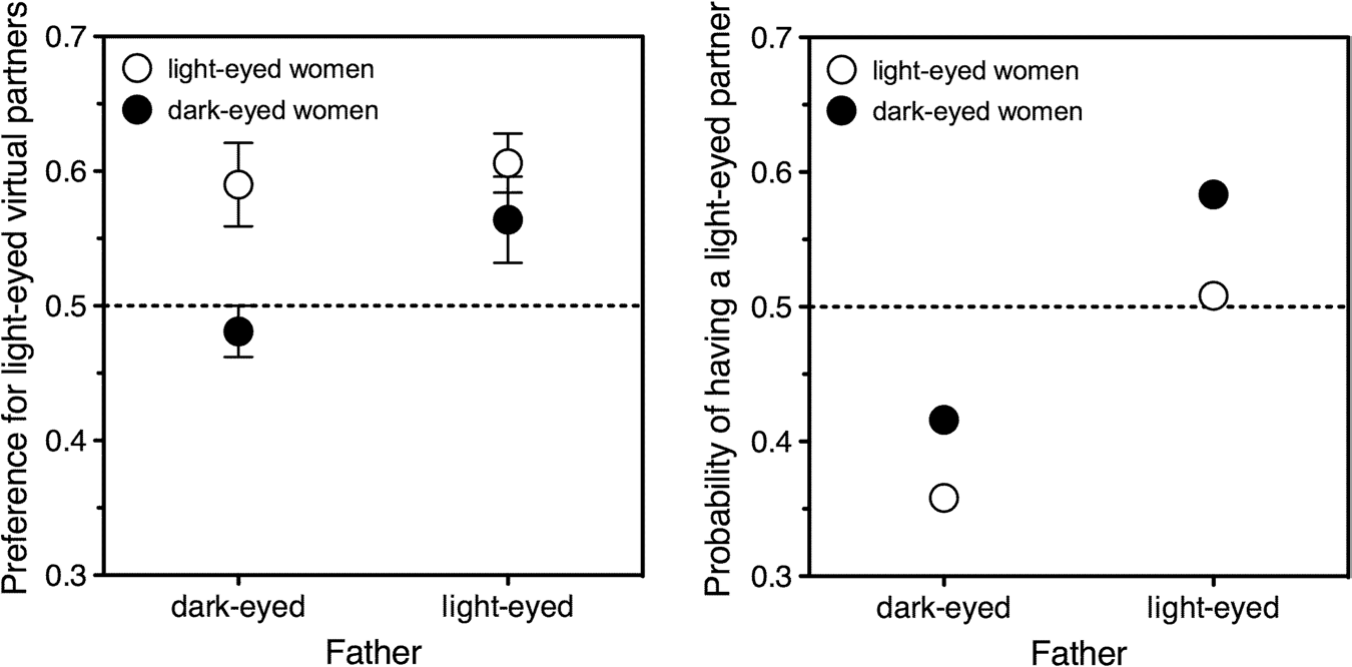
Daughters’ mean preference for light-eyed men as potential long-term partners (left panel) and probability of actually having a light-eyed long-term partner (right panel) as a function of fathers’ eye colour. Data are plotted separately for women with dark and with light eyes. To permit a direct comparison the two panels represent the same women, that is, those who have a real-life partner with either light or dark eyes (*N* = 423). Left panel: Values higher than 0.5 (chance level) represent a preference for light-eyed over dark-eyed men. Error bars indicate one standard error of the mean. Right panel: Values higher than 0.5 (chance level) represent a higher probability of having a light-eyed, as opposed to dark-eyed, partner.

The right panel of Fig. 3 depicts the effect of having a light- or dark-eyed father on the probability of having a light- or dark-eyed partner, separately for light- and dark-eyed women. The left panel shows the virtual-partner preferences expressed by the same women in the long-term context. A comparison between the two panels points to a couple of ways in which unconstrained choices differ from real-life ones. In particular, notice that the effect of own eye lightness that clearly contributed to preferences (compare open symbols with filled symbols) appears to do nothing in real life—means are not even in the expected direction. Second, the same women (daughters of dark-eyed fathers) who expressed either an obvious preference for light-eyed potential partners or no preference whatsoever turn out to be paired with dark-eyed men in real life.

As we had done for the preference data, we tested whether the quality of the relationship between participants and their fathers was associated with the choice of a partner with eyes similar to father’s. Again, the eye colour of fathers correlated with that of partners only for women in the two lower tertiles of paternal rejection (in both cases, *r*_S_ > 0.18, *p* < 0.01) and not in the highest (*r*_S_ = 0.03, *p* = 0.7). Subdivision of participants into tertiles of paternal relationship quality (0–5; 6–8; 9–10), instead of paternal rejection, gave even clearer results—especially when own eye colour was controlled for. Partial correlations were, respectively, *r*_S_ = 0.07, *r*_S_ = 0.12, and *r*_S_ = 0.33. The lowest and highest tertile correlation coefficients are significantly different, *Z* = 2.6, *p* = 0.009, two-tailed.

Current partnership duration in our sample ranged from 15 days to 35 years (mean = 3 years, median = 2.5 years). If only women who had been with their partner longer than one year (indication of an especially stable partnership) were considered, the correlation between father’s and partner’s eye colours (controlling for own eye colour) increased from *r*_S_ = 0.15 to *r*_S_ = 0.17, *p* = 0.0004, *df* = 404. Among the women in the highest tertile of paternal relationship quality, the same correlation rose from *r*_S_ = 0.32 to *r* = 0.42, *p* < 0.0001, *df* = 102.

### Real-partner choice: replication data

In the interests of replicability, we analysed relevant data that had been collected for a different purpose. In this separate study, 340 women (mean age = 23 years, median = 21, range = 18–41 years) filled out an online demographic questionnaire that was in part identical to the one used in the main study. Thus, we obtained information about their own eye colour and that of their parents and partners (194 participants had a partner).

We coded and analysed these additional data in exactly the same way as those of the main study. Eye colour was coded on a 5-point scale from blue to dark brown. Considering mothers, fathers, daughters and partners, we had 165 people with blue, 267 with green, 132 with very-light-brown, 391 with brown, and 194 with dark-brown eyes, for a total of 1149 individuals. The constraint of having cohabited with the relevant parent for longer than 1 year led to no exclusions for analyses involving mothers and to exclusion of 4 participants for analyses involving fathers.

As expected, own eye colour correlated with both maternal (*r*_S_ = 0.34, *p* < 0.0001, *N* = 310) and paternal (*r*_S_ = 0.48, *p* < 0.0001, *N* = 304) eye colours. Again, no evidence of positive assortative mating materialised: neither mothers’ and fathers’ (*r*_S_ = 0.05, *p* = 0.4, *N* = 306) nor participants’ and partners’ (*r*_S_ = −0.03, *p* = 0.7, *N* = 173) eye colours were significantly correlated.

As in the main study, there was no significant association between the eye colours of mothers and partners, *r*_S_ = 0.05, *p* = 0.5, *N* = 174, whereas those of fathers and partners correlated positively and significantly, *r*_S_ = 0.21, *p* = 0.007, *N* = 164 (controlling for own eye colour: *r*_S_ = 0.24, *p* = 0.002, *df* = 161).

### Real-partner choice: combined data

Table 1 shows the number of observed and expected father-partner eye colour pairs in the overall sample (main and replication studies combined). Data refer to our four main colour categories: blue and green (light eyes), brown and dark brown (dark eyes). Participants’ partners with the same eye colour as participants’ fathers were observed more often than would be expected by chance, *χ*^2^(9) = 33.7, *p* = 0.0001. In particular, the combinations blue-eyed father/blue-eyed partner, brown-eyed father/brown-eyed partner, and dark-brown-eyed father/dark-brown-eyed partner all occurred significantly more than expected, whereas the combination dark-brown-eyed father/blue-eyed partner occurred significantly less than expected.

**Table 1:** Distribution of father-partner eye colour pairs. From top to bottom for each colour category: observed frequencies, expected frequencies (within brackets), and adjusted standardised residuals. Standardised residuals above 2 (below −2) suggest that the cell’s observed frequency is significantly greater (smaller) than the expected frequency. Data refer to all the partnered women in our overall sample that reported having cohabited with their father longer than 1 year and whose father’s and partner’s eye colours were either blue, green, brown, or dark brown (*N* = 607).

Fathers	Partners				Total
	Blue	Green	Brown	Dark Brown	
Blue	41	32	40	22	135
	(28.2)	(36)	(45.8)	(24.9)	
	**3**.**1**	−0.9	−1.2	−0.7	
Green	37	43	39	18	137
	(28.7)	(36.6)	(46.5)	(25.3)	
	2	1.4	−1.5	−1.8	
Brown	35	52	88	36	211
	(44.1)	(56.3)	(71.6)	(38.9)	
	−1.9	−0.8	**3**	−0.6	
Dark Brown	14	35	39	36	124
	(25.9)	(33.1)	(42.1)	(22.9)	
	**−3**	0.4	−0.7	**3**.**4**	
Total	127	162	206	112	607

Because of the large size of our combined dataset, we were able to pit the effects of childhood paternal rejection and current paternal relationship quality against one another. To that end, we divided participants into four groups according to whether they were above or below the median in either measurement. Table 2 shows the partial correlations between father’s and partner’s eye colours, controlling for own eye colour, in each of these groups. The correlation failed to reach significance only for women who both had felt rejected by their father as children *and* had a bad relationship with him now. Having felt rejected as a child was not enough to destroy “paternal imprinting” if the current relationship with dad was good, and a poor current relationship was not enough to destroy “paternal imprinting” if the daughter had not felt rejected as a child. These data suggest that paternal influence is robust.

**Table 2:** Effect of daughters’ relationship with their father on the choice of a partner with eyes similar to father’s. Nonparametric partial correlations between father’s and partner’s eye colours, controlling for own eye colour, in the four groups of women created by the combination of low/high scores in our two measures of paternal relationship. Each group’s numerosity is indicated within brackets. Bad/good “current”: participants below/above the median relationship-quality score. Bad/good “childhood”: participants above/below the median rejection score. Asterisks mark statistically significant results. Data refer to all the partnered women in our overall sample that reported having cohabited with their father longer than 1 year and provided both measures of paternal relationship (*N* = 716).

Current	Childhood	
	Bad	Good
Bad	*r*_S_ = 0.08	*r*_S_ = 0.18*
	(*N* = 214)	(*N* = 178)
Good	*r*_S_ = 0.33*	*r*_S_ = 0.24**
	(*N* = 68)	(*N* = 256)

*Correlation is significant, p < 0.02.

**Correlation is significant, p < 0.0002.

## Discussion

We report, for the first time, that having a light-eyed father increases daughters’ preference for light eyes in both a long-term companion and an occasional sex partner. Our two datasets also show that, in real life, women do tend to be paired (long-term) with men whose eye colour resembles their father’s. Both in virtual-partner and actual-partner choices, the effect of paternal eye colour was positively modulated by the quality of the women’s relationship with their father, as assessed by the women themselves.

Having light eyes increased the preference for light-eyed men as potential long-term partners, but not the probability of actually having a light-eyed partner. We found no assortative mating for eye colour between either young couples (our participants and their partners) or older, more established ones (our participants’ mothers and fathers). The strongest correlation in our two Italian samples was *r*_S_ = −0.06.

Given that father-similarity preferences are reflected in actual partner choices, it appears puzzling that self-similarity preferences are not. Current evidence for assortative mating by eye colour is indeed inconsistent, even if one only considers studies with samples larger than 200. No eye-colour correlations have been found between husbands and wives in either Swedish Lapps^23^ or Slovakians^24^. Significant correlations have been reported between husbands and wives of the English “middle classes”^25,26^, in Norwegian couples^27^, and in the participants to an online survey run by a research group based in Scotland^18^. Still, parental effects were controlled in virtually none of these studies, and when they were^18^, women’s assortative mating was shown to be the byproduct of an excess of partners whose eye colour was similar to the women’s fathers’. This might have also been the case in the Norwegian study^27^, which found an excess of partners with the same eye colour as the participants but also an excess of blue-eyed partners for daughters of blue-eyed fathers.

Our findings suggest that women’s preferences for eye colour in a potential long-term partner may result from a “phenotype matching” mechanism that takes, as reference, both own and paternal features. Self-referent matching for facial traits has been shown before. For example, twins like faces that subtly resemble their own better than faces that subtly resemble their (fraternal or even “identical”) co-twin’s^28^. Father-referent matching for facial traits is sexual imprinting by another name. It is possible that facial similarity to the self contributes to a person’s generic likeability (an effect that is already in place in 5-year-olds^29^) whereas facial similarity to the father contributes to a man’s specific attractiveness as a partner. This idea is consistent with our parallel finding that self-resemblance in eye colour fails to increase the attractiveness of short-term partners; a man’s generic likeability would be more important in long- than in short-term relationships.

Having dark eyes and having a dark-eyed father decreased the preference for light-eyed men without ever reversing it into a preference for dark-eyed men (Fig. 2, left panel). At first sight, then, a simple combination of self-referent and father-referent matching falls short of being a complete account of women’s long-term preferences. Yet this pattern of results makes sense in the presence of a superimposed general preference for light eyes. The latter will be expected to counterbalance any latent preference for dark-eyed men—up to the point of cancelling or even reversing it. This hypothesis yields the testable prediction that, in a country where women have no particular preference for light eyes, women with dark eyes and women with dark-eyed fathers should prefer dark-eyed men as virtual long-term partners.

In principle, positive sexual imprinting is not the only possible explanation for the association between fathers’ and partners’ eye colours. Two often-mentioned alternatives can be dismissed right away. The first is familiarity. Mere exposure increases liking^30^. A woman is attracted to men who resemble her parents because she has been exposed to her parent’s faces for a long time. This account requires that maternal traits are liked as much or better than paternal ones, though, because children are normally exposed to their mothers more than to their fathers. Yet we found an effect of paternal, and no effect whatsoever of maternal, eye colour.

The second idea that can be safely set aside is that so-called “imprinting” on opposite-sex parent’s facial traits is a byproduct of assortative mating for the same traits^10^. A woman is attracted to men who resemble her; but because (a) she also resembles her mother and father, and (b) males who resemble her may tend to resemble her father (male) more than they do her mother (female), then (c) she ends up with partners who resemble her father. This account requires resemblance between self and partner as a mediator of resemblance between partner and father, and we found no evidence for this in our sample (see also^31^).

A third option (e.g.^10^) is that daughters might have genetically inherited their mothers’ eye colour preferences. Thus, a mother’s and her daughter’s preference for blue eyes in a mate would create a spurious association between the eye colour of father (mother’s mate) and partner (daughter’s mate). However, it is hard to understand why an inherited preference for a certain eye colour in a partner should be cancelled by a poor relationship with one’s father—which is what we have found. (For a different line of evidence against the inherited-preference explanation, see^31^.) The advantages of learned over inherited mate preferences are discussed in^32^.

A fourth possibility (also mentioned in^10^) is that fathers might try to control their daughters’ choice of a partner, for example by preventing their association with men from a different culture or ethnic group. In 21st century’s Italy that is a bit of a stretch, but even assuming no paternal control, spontaneous assortative mating for ethnicity or culture may well end up producing spurious resemblances between parents and partners. Although it cannot be ruled out in English-speaking samples recruited via the internet, this confound is unlikely to apply within a sample as ethnically and culturally homogeneous^33^ as ours. Within Italy itself, of course, eye colours are not uniformly distributed: the prevalence of light eyes is higher in Northern than in Southern regions and higher in people of Northern than of Southern descent, wherever in Italy they live today. Thus a resemblance between partners’ and parents’ eye colours could still in principle emerge as a side effect of assortative mating for place of residence or ancestry^34^, and thus for eye colour. However, this cannot be the case in our sample, because we found a resemblance between partners’ and fathers’ eye colours *in the absence* of assortative mating for eye colour.

All in all, our data speak to the sexual imprinting explanation much louder than to any alternative hypotheses. By “sexual imprinting” we do not necessarily mean the same process that leads sheep raised by goats to mate with goats later on^3^. For example, it seems that proper sexual imprinting ought to take no account of how good the relationship between offspring and parent is—although we are not aware of any animal literature exploring this issue. Also, sexual imprinting occurs by definition in a limited, sensitive period^35,36^. Our data do not permit us to test this point. We would need a group of women who were exposed to their fathers only *after* early childhood (and extensively from then on, up to adulthood). A literal sexual-imprinting account would predict failure to find paternal effects in such women, whereas a generic, prolonged learning of parental features might operate still.

Sexual imprinting is meant to help the young build a template of what a generic sex partner should look like. This leads to the prediction that men resembling women’s fathers should look more attractive for both long- and short-term relationships. Such a prediction is indeed supported by our data: they suggest that daughters use (good, or at least decent) fathers as models for sexual companions in general.

A stranger’s facial resemblance to a significant other (i.e., to an important person in one’s life, like a close relative or friend) can induce transference^37^. Transference means that the stranger will tend to be unconsciously evaluated as similar in other traits, and as likeable or unlikeable, as the significant other^38,39^. Daughters’ preference for men whose eyes resemble their fathers’ may well be mediated by this mechanism. Still, the characteristics of this transference are far from random. First, eye colour in male faces elicits transference only if it resembles the eye colour of the male, as opposed to female, parent. (Consistently, data on homosexuals suggest that the eye colour of men’s *male* partners tends to resemble that of men’s *male* parent^31^.) Second, this transference is activated by male faces encountered in any mating context, whether or not such a context is consciously associated with parental or life-partner roles. It may be fair to read this particular application of transference as the human rendition of sexual imprinting.
